# Local Synthesis of Estradiol in the Rostral Ventromedial Medulla Protects against Widespread Muscle Pain in Male Mice

**DOI:** 10.1523/ENEURO.0332-24.2024

**Published:** 2024-08-27

**Authors:** Ashley N. Plumb, Joseph B. Lesnak, Louis J. Kolling, Catherine A. Marcinkiewcz, Kathleen A. Sluka

**Affiliations:** ^1^Departments of Physical Therapy and Rehabilitation Science, University of Iowa, Iowa City, Iowa, 52242; ^2^Neuroscience and Pharmacology, University of Iowa, Iowa City, Iowa, 52242

**Keywords:** aromatase, estradiol, estrogen receptor, musculoskeletal pain, testosterone

## Abstract

Animal studies consistently demonstrate that testosterone is protective against pain in multiple models, including an animal model of activity-induced muscle pain. In this model, females develop widespread muscle hyperalgesia, and reducing testosterone levels in males results in widespread muscle hyperalgesia. Widespread pain is believed to be mediated by changes in the central nervous system, including the rostral ventromedial medulla (RVM). The enzyme that converts testosterone to estradiol, aromatase, is highly expressed in the RVM. Therefore, we hypothesized that testosterone is converted by aromatase to estradiol locally in the RVM to prevent development of widespread muscle hyperalgesia in male mice. This was tested through pharmacological inhibition of estrogen receptors (ERs), aromatase, or ER-α in the RVM which resulted in contralateral hyperalgesia in male mice (C57BL/6J). ER inhibition in the RVM had no effect on hyperalgesia in female mice. As prior studies show modulation of estradiol signaling alters GABA receptor and transporter expression, we examined if removal of testosterone in males would decrease mRNA expression of GABA receptor subunits and vesicular GABA transporter (VGAT). However, there were no differences in mRNA expression of GABA receptor subunits of VGAT between gonadectomized and sham control males. Lastly, we used RNAscope to determine expression of ER-α in the RVM and show expression in inhibitory (VGAT+), serotonergic (tryptophan hydroxylase 2+), and μ-opioid receptor expressing (MOR+) cells. In conclusion, testosterone protects males from development of widespread hyperalgesia through aromatization to estradiol and activation of ER-α which is widely expressed in multiple cell types in the RVM.

## Significance Statement

This study's findings reveal, for the first time, that testosterone protects males from development of widespread muscle hyperalgesia through aromatization to estradiol and activation of estrogen receptor alpha (ER-α) within the rostral ventromedial medulla (RVM). The findings also show, for the first time, ER-α expression in the RVM in male mice and the distribution patterns of this expression among multiple cell types. Thus, together our data suggest a unique endogenous mechanism in the RVM for protection against widespread pain in males only.

## Introduction

Women experience more severe, widespread, and longer-lasting chronic pain (Berkley, 1997; Osborne and Davis, 2022) with these differences emerging after puberty suggesting a role for sex hormones in chronic pain (Vincent and Tracey, 2008; King et al., 2011). Research on the sex hormone estrogen shows mixed effects on pain while testosterone consistently protects against pain (Craft et al., 2004; Aloisi et al., 2011; Choi et al., 2012; Lesnak et al., 2020). Clinically, lower testosterone levels are associated with increased pain in rheumatoid arthritis and fibromyalgia and testosterone replacement in these populations decreases pain (Morales et al., 1994; Tengstrand et al., 2009; Pikwer et al., 2014; White et al., 2015; Schertzinger et al., 2018). We developed an animal model of muscle pain that shows unique sexual dimorphism with male mice developing localized pain and female mice developing widespread pain (Gregory et al., 2013). Testosterone mediates this phenotype as gonadectomized males develop widespread pain and testosterone-treated females develop localized pain (Lesnak et al., 2020). These data suggest testosterone is protective in this model; however, the location and mechanisms underlying testosterone's protective effects are unknown.

Preclinical research suggests pain outside the site of insult is mediated by changes in the rostral ventromedial medulla (RVM) and is often referred to as secondary hyperalgesia (Urban et al., 1996, 1999). In rodents, injection of an *N*-methyl-D-aspartate (NMDA) or neurotensin receptor antagonist into the RVM blocks secondary, but not local, hyperalgesia, induced by topical application of mustard oil or intra-articular carrageenan (Urban et al., 1996, 1999). Similarly, development of hyperalgesia in an animal model of chronic widespread pain (repeated acidic saline injection) is prevented by inactivation of the RVM with a local anesthetic, pharmacological blockade of NMDA receptors, or genetic downregulation of the NMDA receptor subunit 1 (NR1; Tillu et al., 2008; Da Silva et al., 2010a,b). Together, these data support a role for the RVM in development of secondary hyperalgesia and widespread pain.

Testosterone can be converted locally to estradiol by the enzyme aromatase which is found in multiple central nervous system regions involved in nociception including the spinal dorsal horn, caudal spinal trigeminal nucleus, and RVM (Schaeffer et al., 2010; Ghorbanpoor et al., 2014; Gao et al., 2017; Tran et al., 2017). Systemic aromatase inhibition in male rodents produces hypersensitivity, enhances ATP-induced pain behaviors, and increases sensory neuron excitability (Robarge et al., 2016), while aromatase inhibition in specific regions of the central nervous system has mixed effects on pain behavior. For example, in the spinal cord and thalamus, inhibition of aromatase increases pain behaviors in animals with neuropathic pain, while aromatase inhibition in the RVM reduces colorectal hyperalgesia with visceral pain (Ghorbanpoor et al., 2014; Gao et al., 2017; Kramer et al., 2018). For estradiol, injection into the RVM or locus ceruleus reduces pain behaviors in male mice injected with formalin (Khakpay et al., 2017), suggesting a protective role in the RVM.

The reduction in pain by aromatase and estradiol in the RVM could involve increased inhibition. Prior studies show estradiol-induced antinociception in the RVM is blocked by GABA_A_ receptor (GABA_A_R) inhibition in the RVM (Khakpay et al., 2017) and aromatase inhibition decreases vesicular GABA transporter (VGAT) expression in the thalamus (Kramer et al., 2018). Because testosterone and RVM are involved in the development of widespread hyperalgesia, we hypothesized that testosterone in RVM is locally synthesized into estradiol to prevent development of widespread pain in males (primary aim). To test this, we pharmacologically inhibited estrogen receptors (ERs) and aromatase in the RVM after induction of muscle pain and then measured muscle hyperalgesia. Further, we genetically deleted ER-α directly in the RVM prior to induction of pain. Lastly, we tested whether testosterone increases expression of GABA receptors and transporters (secondary aim) by quantifying gene expression in gonadectomized and sham control male mice using qPCR and examined localization of ER-α to specific cell types in the RVM using RNAscope.

## Materials and Methods

### Animals

**Figure 1.**
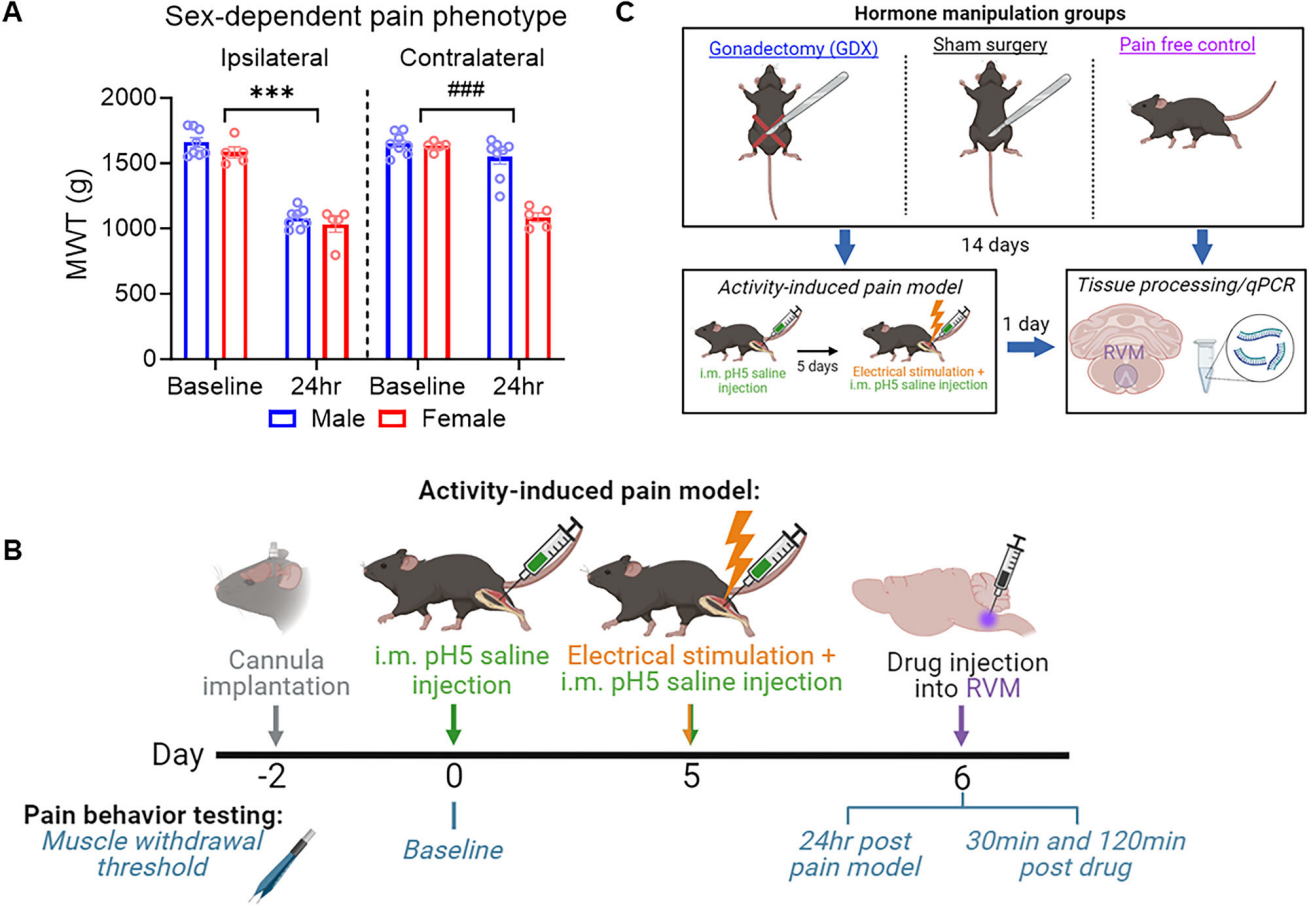
Sex-dependent pain phenotype and experimental design. ***A***, Bar graph showing sex-dependent pain phenotype. After induction of the activity-induced pain model, there was a decrease in MWT in both sexes compared with baseline on the ipsilateral limb. On the contralateral limb, there was a significant decrease in MWT in female but not male mice. ***B***, Timeline for experiments involving microinjection of drugs into the RVM and MWT testing. ***C***, Schematic representation of experimental design of Experiment 2. Figures made using BioRender. See Extended Data Figure 2-1 for ipsilateral data graphs. Data are mean ± SEM with dots representing individual mice. *** significant effect of time *p *< 0.0001; ^###^significant time × sex interaction *p *< 0.0001.

All experiments were approved by the University of Iowa Animal Care and Use Committee and conducted in agreement with the National Institute of Heath guidelines. All animals were housed at the University of Iowa in the animal care facility under a 12 h light/dark cycle with *ad libitum* access to food and water. A total of 131 C57BL/6J mice (120 male, 11 female) and 12 male (Cg)-Esr1tm4.1Ksk/J (*ERα^lox^*) mice (bred in-house) aged between 8 and 16 weeks were utilized. The primary focus of this study was to investigate the protective effects of endogenous testosterone on male mice as shown by the robust behavioral sex differences observed in the activity-induced pain model (Gregory et al., 2013). In this model, males develop unilateral, shorter-lasting muscle hyperalgesia, and females develop bilateral, longer-lasting muscle hyperalgesia as shown again in our vehicle-treated animals in this study (Fig. 1*A*). Because our initial experiments with ER antagonists showed an effect in male but not female mice, all subsequent experiments were exclusively conducted on male animals.

### Animal model of activity-induced muscle pain

The activity-induced pain model uses a combination of intramuscular acidic saline injections with fatiguing muscle contractions as previously described (Gregory et al., 2013). Briefly, mice were anesthetized with 1–5% isoflurane and received a 20 μl intramuscular injection of pH 5.0 ± 0.1 saline into the left gastrocnemius muscle. Similar to intense exercise, this decreases the muscle pH to ∼6.9 (Sluka et al., 2001). Five days following the first intramuscular injection, mice were anesthetized with 1–5% isoflurane followed by insertion of needle electrodes into the left gastrocnemius muscle. The electrical stimulation protocol consisted of three maximal contractions (100 Hz; 7 V pulses at 1 ms duration) followed by 6 min of submaximal contractions (40 Hz, duty cycle of 3.75 s on, 4.25 s off, 90 pulses at 7 V per cycle) and ended with three additional maximal contractions. This protocol leads to an ∼60% decrease in muscle force (Gregory et al., 2013). Immediately following the fatigue protocol, animals received a second intramuscular injection of 20 μl pH 5.0 ± 0.1 saline into the same muscle. Pain-free control mice received a 20 μl injection of pH 7.2 ± 0.1 saline into the left gastrocnemius muscle followed 5 d later by a second 7.2 ± 0.1 saline injection into the same muscle. The combination of muscle insult with muscle fatigue produces long-lasting hyperalgesia in females; however, given alone, muscle insult or electrical stimulation does not produce hyperalgesia (Gregory et al., 2013). Further, this model produces robust behavioral sex differences in which females develop bilateral, longer-lasting hyperalgesia, while males develop localized pain for shorter durations (Fig. 1*A*; Gregory et al., 2013; Lesnak et al., 2020, 2022; 2023; Hayashi et al., 2023).

### Pain behavior testing

Pain behavior was assessed through muscle withdrawal thresholds (MWTs) by applying custom-built force-sensitive tweezers to the gastrocnemius muscle as previously described (Skyba et al., 2005). This measure of muscle hyperalgesia has been previously validated as anesthetizing the skin does not change thresholds, while anesthetizing deep tissue increases thresholds (Skyba et al., 2005). Two days prior to baseline MWT testing, mice were acclimated to a gardener's glove for two, 3–5 min sessions during which their hindlimb was extended outside the glove. During testing, animals underwent three trials in which the gastrocnemius muscle was squeezed with force-sensitive tweezers. Each trial was spaced 5 min apart to avoid sensitization. Both the ipsilateral limb and the contralateral limb of muscle hyperalgesia were tested to assess both local and widespread/secondary muscle hyperalgesia. Increased muscle hyperalgesia is indicated by a lower withdrawal threshold. All testing was completed during the same time of day to account for hormonal fluctuations, and the experimenter was blinded to group assignment during behavioral testing and molecular analysis. Animals were randomly assigned to groups using a computer-generated number.

### Drugs

The following drugs were microinjected into the RVM: (1) ER antagonist (ICI182,780; 0.2–20 µM in 0.9% saline and 0.03% ethanol, 0.2 µl), (2) aromatase inhibitor (letrozole, 350 µM–3.5 mM in 2% DMSO in 0.9% saline, 0.2 µl), (3) ER-α antagonist (MPP dihydrochloride, 10–100 nM in 2% DMSO in 0.9% saline, 0.2 µl), or (4) ER-β antagonist (PHTPP, 10–100 nM in 2% DMSO in 0.9% saline, 0.2 µl; Table 1). The timing and dose of drug were based on previous research (Saleh et al., 2005; Kramer et al., 2018; Kaur et al., 2022). The vehicle control for ER antagonist was 0.9% normal saline and 0.03% ethanol. Vehicle control for the aromatase inhibitor, ER-α antagonist, and ER-β antagonist was 2% DMSO in 0.9% normal saline.

**Table 1.** Pharmacological drugs utilized for RVM microinjection experiments

Pharmacologic	Target	Company	Catalog #	Concentration	Solution
ICI 182,780	ER antagonist; GPER agonist	Tocris Bioscience	1047	0.2–20 µM	0.03% ethanol; 0.9% saline
Letrozole	Aromatase inhibitor	Selleckchem	S1235	350 µM–3.5 mM	2% DMSO; 0.9% saline
MPP	ER-α antagonist	Tocris Bioscience	1991	10–300 nM	2% DMSO; 0.9% saline
PHTPP	ER-β antagonist	Tocris Bioscience	2662	1 µM	2% DMSO; 0.9% saline

### Cannula implantation and stereotaxic intra-RVM injections

Mice were anesthetized with 1–5% isoflurane and placed on a stereotaxic frame for cannula implantation. A 26-guage guide cannula was inserted into the brain based on the stereotaxic coordinates from Paxinos and Franklin Mouse Atlas (Paxinos and Franklin, 2004) for the RVM: AP: −6.2 mm, ML: 0.0 mm, and DV: −5.5 mm. The cannulas were secured with two screws and dental cement. For drug injections, mice were deeply anesthetized with 1–5% isoflurane via vaporization, cannula caps were removed, and a 33-guage injector was attached to a piece of PE20 tubing and a 1 µl Hamilton syringe. Following injection of 0.2 µl of drug, the injector remained in place for 1 min before removal. Animals were then tested for MWT 30- and 120-min post-injection. After cessation of each experiment, cannula placement was verified by injecting 0.2 µl of methylene blue dye into the cannula, followed by transcardial perfusion with heparinized saline and then 4% paraformaldehyde. Injection sites were examined under a light microscope and examined by two blinded investigators to confirm placement. Only animals with injections into the RVM based on the Mouse Brain Atlas were included in the drug groups while injections outside of the RVM were used as missed-site controls (Paxinos and Franklin, 2004).

### Intra-RVM virus injections

*ERα^lox^* mice were deeply anesthetized with 1–5% isoflurane via vaporizer. A craniotomy was performed above the RVM (AP: −6.0 mm from bregma, ML: 0 mm, DV: −5.5 mm from surface). Animals received three 0.2 µl microinjections of AAV8-hSyn-Cre-P2A-dTomato or AAV8-hSyn-mCherry (control) viruses into the RVM spaced 0.1 mm apart at AP: −5.8, −6.0, and −6.2 mm from the bregma, ML: 0.0 mm, DV: −5.5 mm using a 1 µl Hamilton Neuros Syringe (7001 KH, 32 gauge at a rate of 100 nl/min). AAV8-hSyn-Cre-P2A-dTomato was a gift from Ryan Larsen (Addgene viral prep # 107738-AAV8; http://n2t.net/addgene:107738; RRID:Addgene_107738), and AAV8-hSyn-mCherry was a gift from Karl Deisseroth (Addgene plasmid #114472-AAV8; http://n2t.net/addgene:114472; RRID:Addgene_114472). Following each microinjection, the injector was left in place for 10 min then slowly removed. The incision was closed using absorbable sutures and Vetbond (3 M) tissue adhesive. To allow for viral expression, experiments were not performed until 3 weeks following injections.

At the cessation of the experiment, animals were transcardially perfused with heparinized saline followed by 4% paraformaldehyde. Brains were removed and stored in 4% paraformaldehyde overnight, followed by cryoprotection in ascending sucrose solution (10–30%). The tissue was frozen over dry ice in cryomolds filled with optimal cutting temperature compound and then cross sectioned on a cryostat at 20 µm and placed onto slides. To confirm knock-out of the receptor, RVM sections were immunohistochemically stained for ER-α. First, sections were incubated in 5% normal goat serum (VectorLabs, #S-1000) for 30 min followed by an avidin and biotin blocking kit (VectorLabs, #SP-2001). Sections were then incubated in rabbit polyclonal anti-estrogen receptor alpha (1:500; MilliporeSigma, #06-935) primary antibody overnight. On the following day, the slides were washed and then incubated with Biotin-Goat Anti rabbit IgG (1:1,000; Jackson ImmunoResearch, #111-066-144) followed by incubation with Streptavidin Alexa Fluor 488 (1:500; Invitrogen, #A11006) for 1 h each. Slides were then coverslipped with Vectashield with DAPI (VectorLabs; #UX-93952-24) and left at room temperature for 24 h. Stained RVM were then examined on a Leica SP8 confocal microscope in the University of Iowa Microscopy Core to confirm knock-out of ER-α. To count cells, *z*-stacked images containing the RVM were examined using ImageJ software. Using the cell counter tool, an experimenter blinded to treatment group counted the number of cells expressing virus and the number of virus cells coexpressing ER-α. The percent of knockdown was calculated by dividing the virus cells by the ER-α^+^ cells.

### Male gonadectomy surgery

To investigate the impact of testosterone on inhibitory receptors in the RVM, male mice underwent gonadectomy (GDX) or sham gonadectomy (sham) surgery as previously described (Lesnak et al., 2020). Briefly, animals were anesthetized with 1–5% isoflurane. Following a scrotal incision, the testes were exposed, the vas deferens and testicular blood vessels were tied off using synthetic monofilament sutures, and then the testes and epidermis were removed. The incision was closed with silk sutures. We have previously shown that GDX surgery significantly reduces testosterone concentration compared with sham surgery (Lesnak et al., 2020). Sham gonadectomy involved a similar procedure but excluded the ligation or removal of the testes. Animals were then placed in individual cages to protect surgical sites. Pain-free controls were also singly housed during this time. Two weeks after surgery, animals in the gonadectomy and sham control group received the activity-induced pain model followed 24 h later by collection of RVM tissue.

### RNA extraction and qPCR

RVM sections were collected from three groups of male mice: gonadectomized (*n* = 12), sham surgery (*n* = 12), and pain-free control (*n* = 12). To obtain a sufficient RNA yield for qPCR analysis, RVM brain punches from two animals were pooled. Two weeks following surgery, gonadectomized and sham surgery males received the pH 5.0 and fatigue stimuli or control injections. After 24 h, animals were killed with CO_2_. RVM sections collected from punch biopsy were placed in RNA*later* Stabilizing Solution (Invitrogen, Thermo Fisher Scientific, #AM7020) and homogenized, and total RNA was extracted using an RNeasy Lipid Tissue Mini Kit (Qiagen, #74804) followed by purification with an RNA Clean and Concentrate Kit (Zymo Research; #R1013). A NanoDrop One (Thermo Fisher Scientific) was used to measure total RNA concentrations and the ratio of A260/A280 (>1.9 for all samples). Twenty-five nanograms of total RNA from two pooled brains were utilized for cDNA synthesis using an AffinityScript cDNA Synthesis Kit (Agilent Technologies, #600559). Subsequent qPCR was performed using *Power* SYBR Green PCR Master Mix (Applied Biosystems; Thermo Fisher Scientific, #4367659) with custom primers (Integrated DNA Technologies) targeted at GABA_A_R subunits (*Gabra1*, *Gabra2*, *Gabrb2*, *Gabrb3*, *Gabrg1*, and *Gabrg2*), μ-opioid receptor (MOR/*Oprm1*), glutamate ionotropic receptor NMDA type subunit 1 (NR1/*Grin1*), GABA vesicular transporter (VGAT/*Slc32a1*), and κ-opioid receptor (KOR/*Oprk1*; forward and reverse primer sequences listed in Table 2). Primers were designed to ensure specificity to target gene using NCBI Primer-BLAST software (http://www.ncbi.nlm.nih.gov/tools/primer-blast/). Based on preliminary results and previous studies showing high and stable expression, the 36B4 gene was used as an endogenous internal control to normalize the data (Hayashi et al., 2023; Lesnak et al., 2023). The qPCR reactions were performed with the following conditions: 2 min at 50°C, 10 min at 95°C, 40 cycles of 15 s at 95°C, and 1 min at 60°C. All samples were run in triplicate, cycle thresholds were averaged between the three samples, and relative quantification of gene expression was analyzed with the QuantStudio 7 Flex Real-Time PCR System (Applied Biosystems; Thermo Fisher Scientific) using the 2^−ΔΔCt^ method.

**Table 2.** Primer sets utilized in quantitative polymerase chain reaction

Gene name	Accession number		Primer sequence (5′–3′)	Product size
*Gabra1*	NM_001359035.1	F	GTTCTAGCAGGGAAGCGAGCA	137 bp
		R	TTCCCGACAGTGTGCTCAGAA	
*Gabra2*	NM_008066.4	F	TCTTTGCCTCATTCAGCTGCC	94 bp
		R	TGAAGCTGTGACCCTGGTATGAG	
*Gabrb2*	NM_001347314.3	F	GTGGCAGACCAACTCTGGGT	155 bp
		R	ATCATGCAGGCAGCCGTAGT	
*Gabrb3*	NM_008071.3	F	TTTCGGCATCTTCTCGGCCC	192 bp
		R	TCGATGCTGGCGATGTCGAT	
*Gabrg1*	NM_010252.5	F	GCAGCACTCATGGAATACGGA	118 bp
		R	ATCCAGCATGGAGACCTGGG	
*Gabrg2*	NM_008073.4	F	CACCGGGCATGAATGTGAGC	83 bp
		R	GAGGCAGGGGGATGGTACAC	
*Oprm1*	NM_001302793.1	F	GCTCAGACGTTCCATTCTGCC	172 bp
		R	TGAATGCTTGCTGCGGACTC	
*Grin1*	NM_008169.3	F	AGTTCATGTGGTGGCCGTGA	194 bp
		R	ACGAGCAGAGAAACTCCGGG	
*Slc32a1*	NM_009508.2	F	CATGCTGCGCTCCTCGATTC	178 bp
		R	AGCACCGCTGTGGCTATGAT	
*Oprk1*	NM_011011.2	F	CGCACCTTGCTGATCCCAAA	90 bp
		R	TTCAGCCCTGATGGGTCCAC	
*36B4* (House Keeping)	NM_007475.5	F	GCAGGTGTTTGACAACGGCA	190 bp
		R	CACAGACAATGCCAGGACGC	

### In situ hybridization

To determine what cell types express ER-α in the RVM, we used RNAscope to examine ER-α expression on inhibitory (VGAT+), serotonergic (TPH2+), and μ-opioid receptor (MOR+) positive cells. Naive mice were perfused with 0.9% heparinized normal saline and then perfused with 4% paraformaldehyde. Brains were extracted, stored in 4% paraformaldehyde overnight, cryopreserved in 10–30% sucrose, and frozen in OCT (Thermo Fisher Scientific) in a cryomold. Tissues were stored at −20°C until processing. Coronal sections containing RVM regions were cut on a cryostat (−20°C) and mounted to Superfrost Plus Frosted Microscope Slides (Fisher Scientific; #1255015) and then stored at −20°C until further processing. For in situ hybridization, RNAscope Fluorescent V2 Multiplex Assay (ACDBio; #323270) was used following the ACDBio protocol. Briefly, slides were baked at 60°C, postfixed in chilled 10% neutral buffered formalin, and then dehydrated in increasing concentrations of ethanol solution (50%, 70%, 100%, 100%). RNAscope Hydrogen Peroxide was then applied, and target retrieval was performed using a steamer. A hydrophobic barrier was drawn around each section, and sections were incubated in RNAscope Protease III. The slides were then washed and incubated in HybEZ oven for 2 h at 40°C with experimental probes: ER-α receptor (ER-α/ESR1; ACD 478201 – C2), μ-opioid receptor (MOR/*Oprm1*; ACD 315841 – C1), vesicular Gaba transporter (VGAT/*Slc32a1*; ACD 319191 – C3), positive control probes: ubiquitin C (UBC), peptidyl-prolyl *cis*-trans isomerase B (PPIB), and DNA-directed RNA polymerase II subunit RPB1(POLR2A) or a negative control probe: bacterial gene dihydrodipicolinate reductase (DapB). In a separate experiment, probes targeted at ER-α receptor (ER-α/*ESR1*; ACD 478201 – C3) and tryptophan hydroxylase 2 (*Tph2*; ACD 318691 – C2) were applied to tissue sections. Following incubation, slides were washed and then placed in 5× SCC buffer overnight. Amplification steps were performed the following day using AMP-1, AMP-2, and AMP-3 followed by sequential treatment with TSA Vivid 520, 570, and 650 Dyes (1:1,500; Akoya Biosciences). Slides were coverslipped with Vectashield with DAPI (VectorLabs; #UX-93952-24) and air-dried overnight at room temperature. Images were captured at 20× magnification using an Olympus VS200 slide scanner and analyzed using QuPath 0.4.3 software. Cell detection software was applied to each section (3–4 sections per mouse across eight mice), and only cells with nuclei visible by DAPI staining were counted. For quantification, percent coexpression was calculated for cells containing ER-α receptor.

### Experimental design

#### Experiment 1

In this experiment, we aimed to investigate whether the protective effects of testosterone on muscle pain in male mice are mediated by the aromatization of testosterone to estradiol and the activation of ER in the RVM (Fig. 1*B*). (1a) To confirm the role of ERs in the RVM, male and female mice were microinjected with an ER antagonist (ICI-182,780) into the RVM. (1b) To determine whether this ER activation was from local testosterone conversion to estradiol in the RVM, male mice were microinjected with an aromatase inhibitor (letrozole) into the RVM. (1c) To determine which ER was involved, male mice were microinjected with specific ER-α antagonist (MPP dihydrochloride) or ER-β antagonist (PHTPP). For each of these experiments, mice received microinjection of the drug being tested into the RVM 24 h after induction of the pain model, and MWTs were assessed at baseline, 24 h after induction of the pain model, and again 30 and 120 min after drug injection. (1d) Lastly, because specific ER antagonists exhibit concentration-specific nonspecificity (Kaur et al., 2022), we genetically ablated ER-α using *ERα^lox^* mice and injection of AAV8-hsyn-Cre-P2A-dTomato. Control mice received AAV8-hsyn-mcherry into the RVM. Three weeks after viral-mediated ER-α knock-out, the pain model was induced. MWTs were assessed prior to induction of the pain model and again 24 h postinduction of the pain model.

#### Experiment 2

This experiment aimed to assess whether systemic testosterone modulates expression of inhibitory receptors and transporters in the activity-induced pain model using gonadectomized (GDX), sham, and naive male mice. GDX or sham surgery was performed 2 weeks prior to induction of the pain model as previously described (Lesnak et al., 2020). Brain tissue was collected 24 h after induction of the pain model, and qPCR analysis was completed on the RVM tissue with primers targeted at GABA_A_R subunits (*Gabra1*, *Gabra2*, *Gabrb2*, *Gabrb3*, *Gabrg1*, and *Gabrg2*), VGAT/*Slc32a1*, MOR/*Oprm1*, NR1/*Grin1*, and KOR/*Oprk1* (Fig. 1*C*). These GABA_A_R subunits were chosen because they make up the majority of GABA_A_R (Sieghart et al., 1999), some of which are modulated by gonadectomy (Agrawal and Dwivedi, 2020). VGAT was chosen since it is modulated by aromatase inhibition in the thalamus (Kramer et al., 2018). Lastly, MOR, NR1, and KOR were chosen as each are widely implicated in pain modulation in the RVM (Da Silva et al., 2010b; Brito et al., 2017; Nguyen et al., 2022).

#### Experiment 3

In this experiment, we investigated whether ER-α is colocalized with putative markers of cells in the RVM in male mice. Classically, RVM neurons are classified into ON, OFF, and NEUTRAL cells which can be identified through their electrophysiological response to nociceptive stimulation. ON cells facilitate nociception, express μ-opioid receptors (MORs), and are directly inhibited by MOR agonist which are antinociceptive (Barbaro et al., 1986; Heinricher et al., 1994). Activation of GABAergic (VGAT+) neurons in the RVM can facilitate pain behavior (Francois et al., 2017). Serotonergic neurons are classically characterized as neutral cells (Potrebic et al., 1994); however, recent evidence suggests that TPH2 is expressed on a subset of OFF cells and 5-HT injected into the RVM is antinociceptive (Llewelyn et al., 1983; Winkler et al., 2006). Thus, we used RNAscope to localize expression of ER-α (*Esr1*) in the RVM with VGAT (*Slc32a*) as a marker for GABAergic neurons and μ-opioid receptors (MOR/*Oprm1*) as a marker of ON-cells. The number of different cell types (*Esr1*, *Esr1 *+ *Slc32a*, *Esr1 *+ *Oprm1*, and *Esr1 *+ *Slc32a *+ *Oprm1*) was quantified. In a separate experiment, we analyzed expression of ER-α (*Esr1*) on TPH2+ cells to examine expression on serotonergic cells.

### Statistical analysis

Statistical analyses were performed using SPSS Version 25.0 (IBM SPSS). The sample sizes calculation for the primary outcome measure was determined utilizing a power of 0.8, a significance threshold of 0.05, and effect sizes derived from preliminary data. Specifically, for the primary outcome measure, a sample size of eight animals per group was determined which showed 80% statistical power at *p* < 0.05 (*F*_(1,5) _= 6.870; *pᶯ*^2 ^= 0.579 for ER antagonist). For qPCR, an *n* of six animals per group was used based on previous data published from our laboratory, which has previously been shown to be sufficient to detect differences in RVM staining and mRNA expression between groups (Bobinski et al., 2015; Lesnak et al., 2020). To confirm sex differences in behavioral data as we have previously shown in this pain model, we used a repeated-measures ANOVA with Tukey's post hoc test on baseline and 24 h MWT data from male and female vehicle control mice. All other behavioral data was analyzed with one-way ANOVA or *t* test on the change score (24 h post model and 30 min post drug injection, results in Table 3) followed by Tukey’s post hoc test where appropriate (post-hoc results in Table 4, sample sizes in Table 5). Statistical analysis for qPCR data was analyzed with a one-way ANOVA to test differences between conditions (gonadectomized, sham, and pain-free control) followed by a post hoc Tukey’s test to analyze individual group differences. For immunohistochemistry data, a *t* test was competed to compare differences in expression of ER-α between virus and control. No statistical analysis was completed on the in situ hybridization.

**Table 3.** Statistical analysis results for behavioral pharmacology on both the ipsilateral and contralateral side (one-way ANOVA or *t* test on the change score)

Drug	Limb	Effect for group	*p* value
ICI 182,780 (male)	Ipsilateral	*F*_(4,30) _= 1.36	*p *= 0.271
	Contralateral	*F*_(4,30) _= 5.6	*p *= 0.002
ICI 182,780 (female)	Ipsilateral	*t*_(9) _= 0.15	*p *= 0.882
	Contralateral	*t*_(9) _= 1.22	*p *= 0.254
Letrozole	Ipsilateral	*F*_(4,19) _= 0.09	*p *= 0.985
	Contralateral	*F*_(4,19) _= 8.0	*p *= 0.0006
PHTPP	Ipsilateral	*t*_(7) _= 0.52	*p *= 0.619
	Contralateral	*t*_(7) _= 0.10	*p *= 0.922
MPP	Ipsilateral	*F*_(2,15) _= 2.09	*p *= 0.195
	Contralateral	*F*_(2,15) _= 13.78	*p *= 0.0004

**Table 4.** Tukey's post hoc results for behavior studies

	
ICI182,780 (Tukey’s post hoc)	
Veh vs 0.2 µM	*p *= 0.051
Veh vs 2 µM	*p *= 0.033
Veh vs 20 µM	*p *= 0.004
Veh vs missed site	*p *= 0.998
Missed site vs 20 µM	*p *= 0.032
Letrozole doses (Tukey’s post hoc)	
Veh vs 350 µM	*p *= 0.327
Veh vs 1 mM	*p *= 0.022
Veh vs 3.5 mM	*p *= 0.001
Veh vs missed site	*p *= 0.982
Missed site vs 3.5 mM	*p *= 0.003
MPP doses (Tukey’s post hoc)	
Control vs 300 nM	*p *= 0.0006
Control vs 10 nM	*p *= 0.958
10 vs 300 nM	*p *= 0.004

**Table 5.** Sample size for behavioral studies

Drug	Dose	Sample size
ICI 182,780 (male)	Vehicle	8
	Missed site	5
	0.2 µM	7
	2 µM	7
	20 µM	8
Letrozole (male)	Vehicle	5
	Missed site	5
	350 µM	4
	1 mM	5
	3.5 mM	5
PHTPP (male)	Vehicle	5
	Drug	4
MPP (male)	Vehicle	7
	10 nM	4
	300 nM	7

## Results

### Activity-induced muscle pain phenotype

To confirm the previously identified sex differences in our pain model, we analyzed data from vehicle-treated animals in Experiment 1a before and 24 h after induction of the activity-induced pain model (prior to injection into the RVM). After induction of the activity-induced pain model, there was a significant time × side × sex interaction (*F*_(1,22) _= 25.47; *p *< 0.0001; repeated-measures ANOVA). Therefore, separate repeated-measures ANOVA analysis for both the ipsilateral and contralateral limbs were conducted to examine sex differences. On the ipsilateral limb, there was a significant difference in MWT between baseline and 24 h (*F*_(1,11) _= 475.33; *p *< 0.0001; repeated-measures ANOVA) but no time by sex interaction (*F*_(1,11) _= 0.31; *p *= 0.5910; repeated-measures ANOVA; Fig. 1*A*). Therefore, after induction of the model, both male and female mice show a significant decrease in MWT on the ipsilateral limb. On the contralateral limb however, there was a significant difference in MWT between baseline and 24 h (*F*_(1,11) _= 70.64; *p *< 0.0001; repeated-measures ANOVA) and a significant time by sex interaction (*F*_(1,11) _= 32.61; *p *< 0.0001; repeated-measures ANOVA) with females showing a significant decrease in MWT compared with males (Fig. 1*A*). This is similar to prior studies (Gregory et al., 2013; Lesnak et al., 2022) showing a bilateral decrease in MWT 24 h after induction of the model in female mice and a unilateral decrease in male mice.

**Figure 2.**
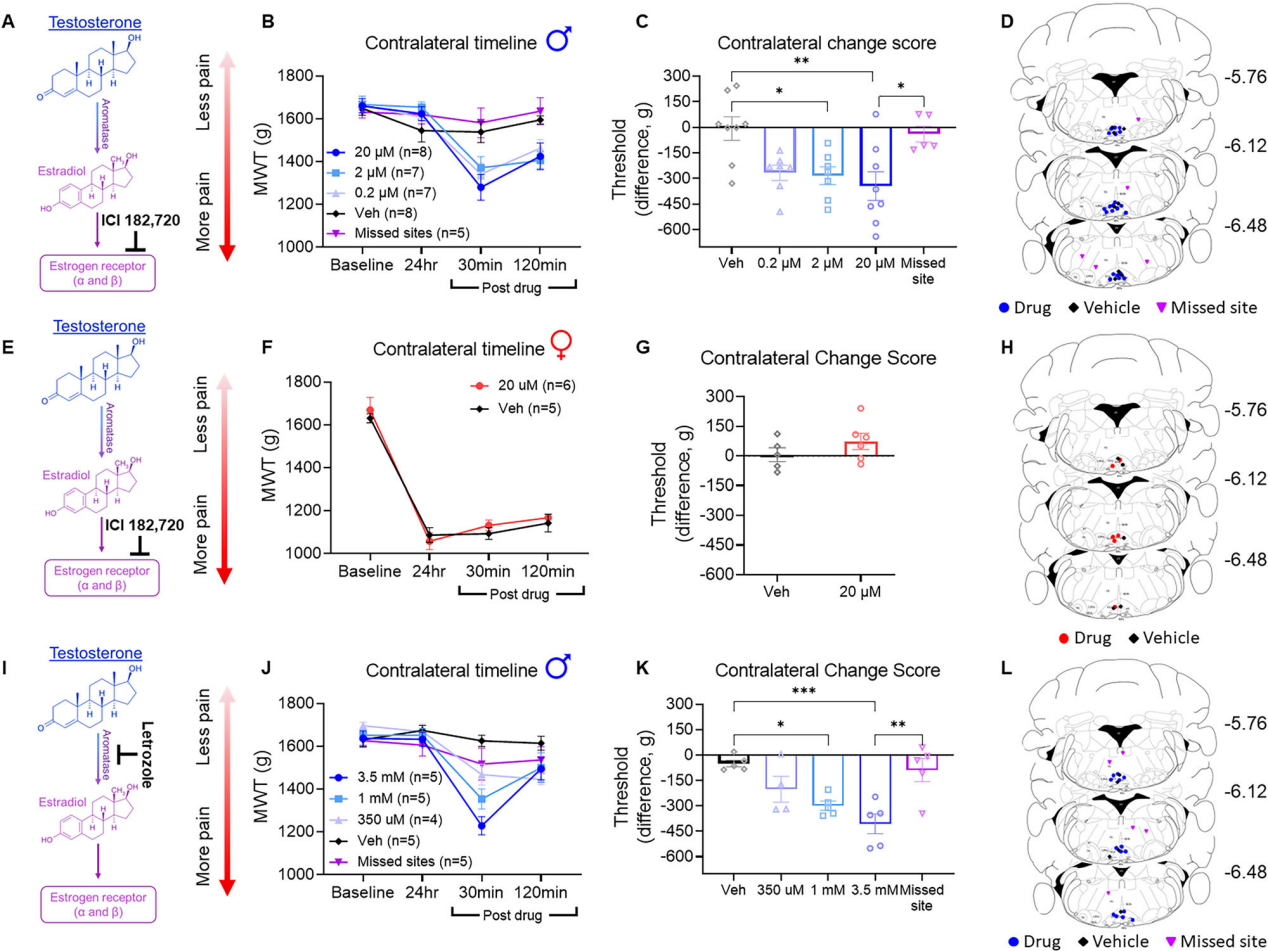
Inhibition of ERs and estradiol synthesis in the RVM produces widespread hyperalgesia in male mice. ***A***, Schematic representation showing the action ER antagonist, ICI 182,780, which antagonizes both ER-α and ER-β. ***B***, Line graph displaying the change in MWT scores in males, demonstrating a decrease at 30 min postmicroinjection of ICI 182,780, followed by a partial recovery at 120 min postdrug administration. ***C***, Male mice show a significant difference in change scores when comparing MWT at 24 h post pain model to 30 min postdrug injection of 2 and 20 µM of ICI 182,780 compared with vehicle. ***D***, Location map of cannula placements for ICI 182,780, vehicle, and missed placed cannula sites in male mice. ***E***, Schematic representation showing the action ER antagonist, ICI 182,780, which antagonizes both ER-α and ER-β. ***F***, Line graph showing MWT scores in female mice, indicating no difference at the 30 or 120 min marks postinjection of ICI 182,780. ***G***, Female mice show no significant difference in MWT change scores between vehicle and 20 µm of ICI 182,780. ***H***, Location map of cannula placements for ICI 182,780, vehicle, and missed placed cannula sites in female mice. ***I***, Schematic representation showing action of the aromatase inhibitor letrozole, which inhibits the conversion of testosterone to estradiol. ***J***, Line graph displaying the change in MWT scores over time in male mice, showing a decrease at 30 min postmicroinjection of letrozole, followed by a partial recovery at 120 min postdrug administration. ***K***, Male mice show a significant difference in change scores when comparing MWT at 24 h postpain model to 30 min postdrug injection of 1 or 3.5 mM letrozole compared with vehicle. ***L***, Location map of cannula placements for letrozole, vehicle, and missed placed cannula sites in male mice. See Extended Data Figure 2-1 for ipsilateral data graphs. Data are mean ± SEM with dots representing individual mice; *p < 0.05, **p < 0.01 ***p < 0.005 indicate statistical significance.

10.1523/ENEURO.0332-24.2024.f2-1 Figure 2-1 Inhibition of ERs, estradiol synthesis, ER-α, or ER-β in the RVM or genetic deletion of ER-α has no impact on ipsilateral hyperalgesia. Download Figure 2-1, TIF file.

### Inhibition of ERs and estradiol synthesis in the RVM produces widespread hyperalgesia in male mice

To investigate the role of ER in the RVM on protection from development of widespread pain in male mice, we microinjected an ER antagonist (ICI 182,780; Fig. 2*A–H*) 24 h after induction of the pain model. Inhibition of ER in the RVM resulted in a significant dose-dependent decrease in MWT change score on the contralateral limb (*F*_(4,30) _= 5.6; *p *= 0.002; one-way ANOVA; Fig. 2*B*,*C*) with significant differences between groups receiving 2 µM (*p *= 0.033; Tukey's test) or 20 µM of ICI 182,780 (*p *= 0.004; Tukey's test) compared with vehicle control group (Fig. 2*C*). However, mice that received 20 µM drug injection outside the RVM showed no significant differences compared with vehicle control (*p *= 0.998; Tukey's test). There were no significant differences in MWT change score between groups on the ipsilateral limb in male mice (*F*_(4,30) _= 1.36; *p *= 0.271; one-way ANOVA; Extended Data Fig. 2-1*A,B*). Further, there were no significant differences in MWT change score between groups on either the ipsilateral (*t*_(9) _= −0.15; *p *= 0.882; *t* test; Extended Data Fig. 2-1*C,D*) or contralateral limb (*t*_(9) _= 1.22; *p *= 0.254; *t* test; Fig. 2*F*,*G*) in female mice. Injection sites for animals in each group including drug, vehicle, and missed-site controls are shown in Figure 2*D*,*H*.

Because aromatase can locally convert testosterone to estradiol, we tested if administration of an aromatase inhibitor (letrozole; Fig. 2*I–L*) in the RVM produced widespread pain in male mice. Specifically, inhibition of aromatase (letrozole) in the RVM resulted in a dose-dependent decrease in MWT change score on the contralateral limb (*F*_(4,19) _= 8.0; *p *= 0.0006; one-way ANOVA; Fig. 2*J*,*K*), with significantly lower MWT in animals that received 1 or 3.5 mM letrozole compared with vehicle control group (*p *< 0.05; Tukey's test; Fig. 2*K*) but not between drug injection outside the RVM compared with vehicle control (*p *= 0.98; Tukey's test). There were no significant differences in MWT changes score between drug and control groups on the ipsilateral limb (*F*_(4,19) _= 0.09; *p *= 0.985; one-way ANOVA; Extended Data Fig. 2-1*E,F*). Injection sites for those in the drug, vehicle and missed-site group are shown in Figure 2*L*.

### ER-α but not ER-β protects male mice from the development of widespread pain

**Figure 3.**
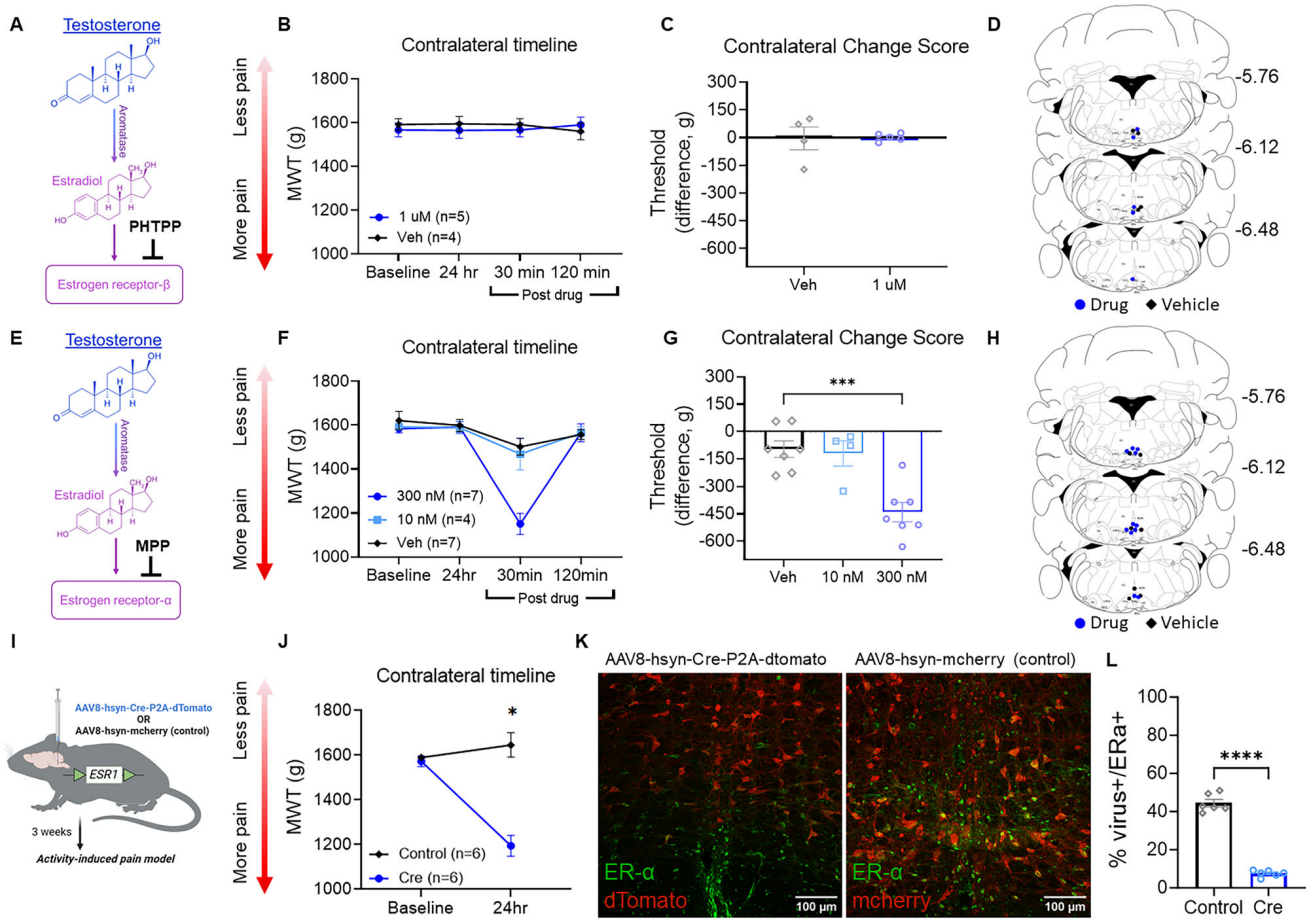
ER-α but not ER-β protects male mice from the development of widespread pain. ***A***, Schematic representation showing the action of ER-β antagonist PHTPP. ***B***, Line graph showing MWT scores in male mice, indicating no difference at the 30 or 120 min marks postinjection of PHTPP. ***C***, Male mice show no significant difference in MWT change scores between vehicle and 1 μm of PHTPP. ***D***, Location map of cannula placements for PHTPP, vehicle, and missed placed cannula sites in male mice. ***E***, Schematic representation showing the action of ER-α antagonist MPP. ***F***, Line graph displaying the change in MWT scores over time in male mice, showing a decrease at 30 min postmicroinjection of MPP, followed by a recovery at 120 min postdrug administration. ***G***, Male mice show a significant difference in change scores when comparing MWT at 24 h postpain model to 30 min postdrug injection of 300 nM MPP compared with vehicle. ***H***, Location map of cannula placements for MPP, vehicle, and missed placed cannula sites in male mice. ***I***, BioRender image showing injection of virus into the RVM of *ERα^lox^* mice. ***J***, Line graph of MWT at baseline and 24 h postinduction of pain model. *ERα^lox^* mice that received the Cre virus show a significant decrease in contralateral MWT 24 h postinduction of pain model compared with controls. ***K***, Validation of the viral approach to knock-out ER-α in the RVM. Immunostaining for ER-α confirms knock-out of ER-α expressing neurons in mice that received the cre virus (left) compared with the control virus (right). ***L***, Quantification of ER-α^+^ cells illustrate a significant decrease in the number of ER-α^+^ in mice that received Cre versus control virus. Data are mean ± SEM with dots representing individual mice; *p < 0.05 and ****p < 0.0001 indicate statistical significance. Graphical representations of experiments were made using BioRender.

To determine which ER was involved in this protection against development of widespread pain in male mice, we microinjected either a selective ER-β (PHTPP; Fig. 3*A–D*) or ER-α (MPP; Fig. 3*E–H*) antagonist into the RVM. No significant differences in MWT change scores were observed on the contralateral limb between mice that received PHTPP (ER-β antagonist) compared with mice receiving vehicle (*t*_(7) _= 0.10; *p *= 0.922; *t* test; Fig. 3*B*,*C*). In contrast, MWT change scores on the contralateral limb were significantly different after microinjection of MPP (ER-α antagonist) into the RVM (*F*_(2,15) _= 13.78; *p *= 0.0004; *t* test; Fig. 3*F*,*G*) with Tukey post hoc analysis revealing a significant difference between 300 nM dose of MPP compared with vehicle control (*p *< 0.01). There were no significant differences in MWT change score between groups on the ipsilateral limb after microinjection of MPP (*F*_(2,15) _= 2.09; *p *= 0.159; one-way ANOVA; Extended Data Fig. 2-1*G,H*) or PHTPP (*t*_(7) _= 0.52; *p *= 0.619; *t* test; Extended Data Fig. 2-1*I,J*).

Since inhibition of ER-α in the RVM decreased MWT in male mice, we investigated whether genetic deletion of ER-α in the RVM prior to induction of the pain model would impact the development of muscle pain (Fig. 3*I–L*). *Erα^lox^* mice were microinjected with either AAV8-hsyn-Cre-P2A-dTomato or AAV8-hsyn-Cre-mcherry (control) viruses 3 weeks prior to induction of the pain model and tested for pain at baseline and 24 h postinduction of the pain model. As expected, mice treated with AAV8-hsyn-Cre-P2A-dTomato showed a significant reduction in ER-α immunohistochemical staining in the RVM (Fig. 3*K*,*L*) when compared with controls injected with AAV8-hsyn-mcherry (*t*_(10) _= 18.10; *p *< 0.0001; *t* test). There was a significant group difference in the MWT on the contralateral side between mice that received AAV8-hsyn-Cre-P2A-dTomato compared with AAV8-hsyn-Cre-mcherry (control) virus (*t*_(10) _= 6.22; *p *< 0.0001; *t* test; Fig. 3*J*) with mice that received Cre virus showing lower MWT compared with control virus. There was no difference in MWT in mice that received AAV8-hsyn-Cre-P2A-dTomato compared with AAV8-hsyn-Cre-mcherry (control) prior to induction of the model (*t*_(10) _= 0.56; *p *= 0.585; *t* test; Fig. 3*J*) or postinduction on the ipsilateral side (*t*_(10) _= 0.14; *p *= 0.888; *t* test; Extended Data Fig. 2-1*K,L*).

### Testosterone does not alter mRNA expression of GABA_A_ receptor subunits and VGAT in the RVM

**Figure 4.**
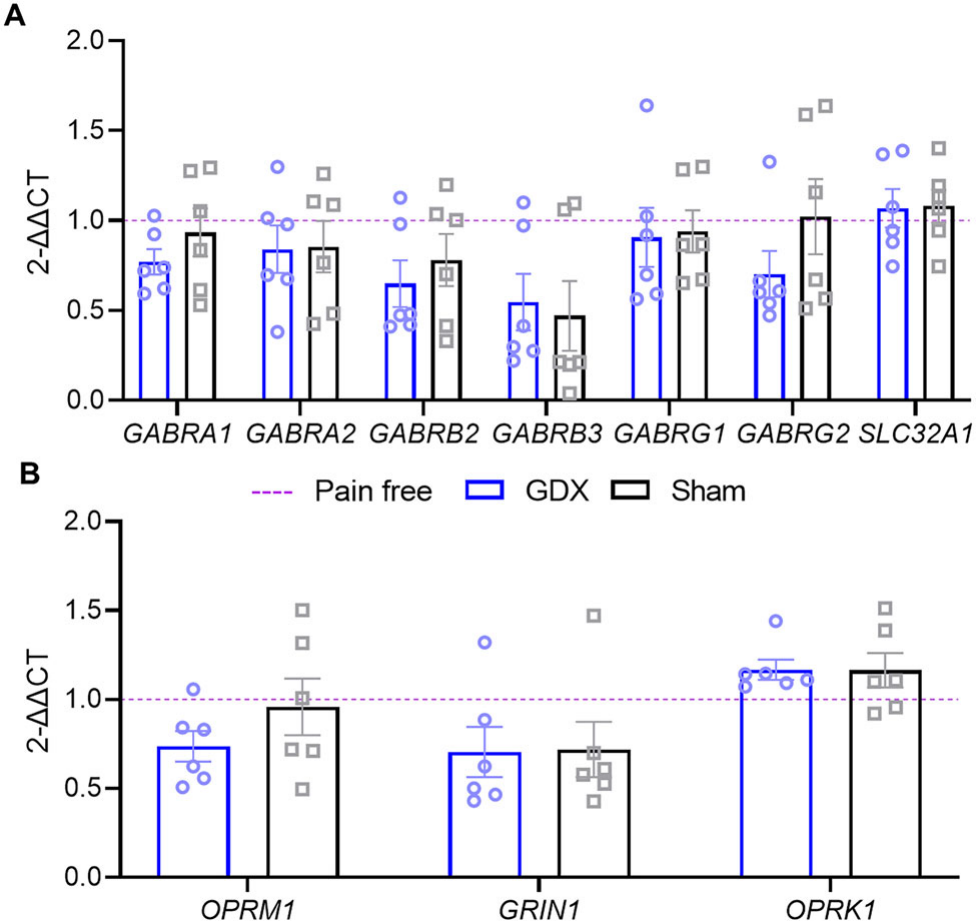
Inhibitory receptors are unaltered by reduced testosterone prior to muscle pain. ***A***, qPCR analysis of GABA_A_R and VGAT (*Slc32a1*) genes compared with pain-free, gonadectomized (GDX), and sham control male mice. ***B***, qPCR analysis of *Oprm1*, *Grin1*, and *Oprk1* compared with pain-free (*n* = 6), gonadectomized (GDX; *n* = 6), and sham control (*n* = 6) male mice. Figures were made using BioRender.

In other brain regions, ER agonists modulate expression of receptors involved in pain and analgesia including GABA_A_R subunits, MOR, and NMDA receptor subunits (Herbison and Fénelon, 1995; Gazzaley et al., 1996; Quiñones-Jenab et al., 1997). We used qPCR to quantify mRNA expression of GABA_A_R subunits (*Gabra1*, *Gabra2*, *Gabrb2*, *Gabrb3*, *Gabrg1*, and *Gabrg2*) and GABA vesicular transporters (VGAT/*Slc32a1*), our primary aim for the qPCR experiments, in the RVM following induction of the activity-induced pain model in gonadectomized, sham control, and pain-free animals. GABA_A_R subunit mRNA expression (*Gabra1*, *Gabra2*, *Gabrb2*, *Gabrg1*, and *Gabrg2*) was not different between groups (*F*_(2,15) _= 0.65–3.77; *p *= 0.129–0.536; one-way ANOVA; Fig. 4*A*; detailed statistical analysis results listed in Table 6) except for *Gabrb3* (*F*_(2,15) _= 0.05; *p *= 0.047; one-way ANOVA). However, after controlling for multiple comparisons (*p* = 0.017), there was no significant difference in *Gabrb3* mRNA levels between groups [gonadectomized vs sham (*p *= 0.93), gonadectomized vs pain-free (*p *= 0.11), or sham vs pain-free (*p *= 0.06); Tukey's test]. There was also no significant difference between groups for *Slc32a1* (VGAT) mRNA (*F*_(2,15) _= 0.06; *p *= 0.938; one-way ANOVA; Fig. 4*A*). We also examined mRNA expression of glutamate ionotropic receptor NMDA type subunit-1 (NR1/*Grin1*), μ-opioid receptor (MOR/*Oprm1*), and κ-opioid receptor (KOR/*Oprk1*). There were no significant differences in mRNA expression between groups for *Grin1* (NR1), *Oprm1* (MOR), and *Oprk1* (KOR; *F*_(2,15) _= 1.10–1.77; *p *= 0.204–0.358; one-way ANOVA; Fig. 4*B*; detailed statistical analysis results listed in Table 4). These findings suggest that reducing testosterone levels prior to hyperalgesia does not impact the mRNA levels of these inhibitory receptors in the RVM.

**Table 6.** Statistical analysis results for quantitative polymerase chain reaction (one-way ANOVA comparing pain-free, sham, and gonadectomized male mice)

Gene name	*F*_(DFn,DFd)_	*p* value
*Gabra1*	*F*_(2,15) _= 1.56	*p *= 0.243
*Gabra2*	*F*_(2,15) _= 0.65	*p *= 0.536
*Gabrb2*	*F*_(2,15) _= 2.37	*p *= 0.128
*Gabrb3*	*F*_(2,15) _= 3.77	*p *= 0.047
*Gabrg1*	*F*_(2,15) _= 1.06	*p *= 0.371
*Gabrg2*	*F*_(2,15) _= 1.52	*p *= 0.249
*Oprm1*	*F*_(2,15) _= 1.51	*p *= 0.253
*Grin1*	*F*_(2,15) _= 1.77	*p *= 0.204
*Slc32a1*	*F*_(2,15) _= 0.06	*p *= 0.938
*Oprk1*	*F*_(2,15) _= 1.10	*p *= 0.358

### A majority of ER-α is colocalized with VGAT and MOR positive cells

**Figure 5.**
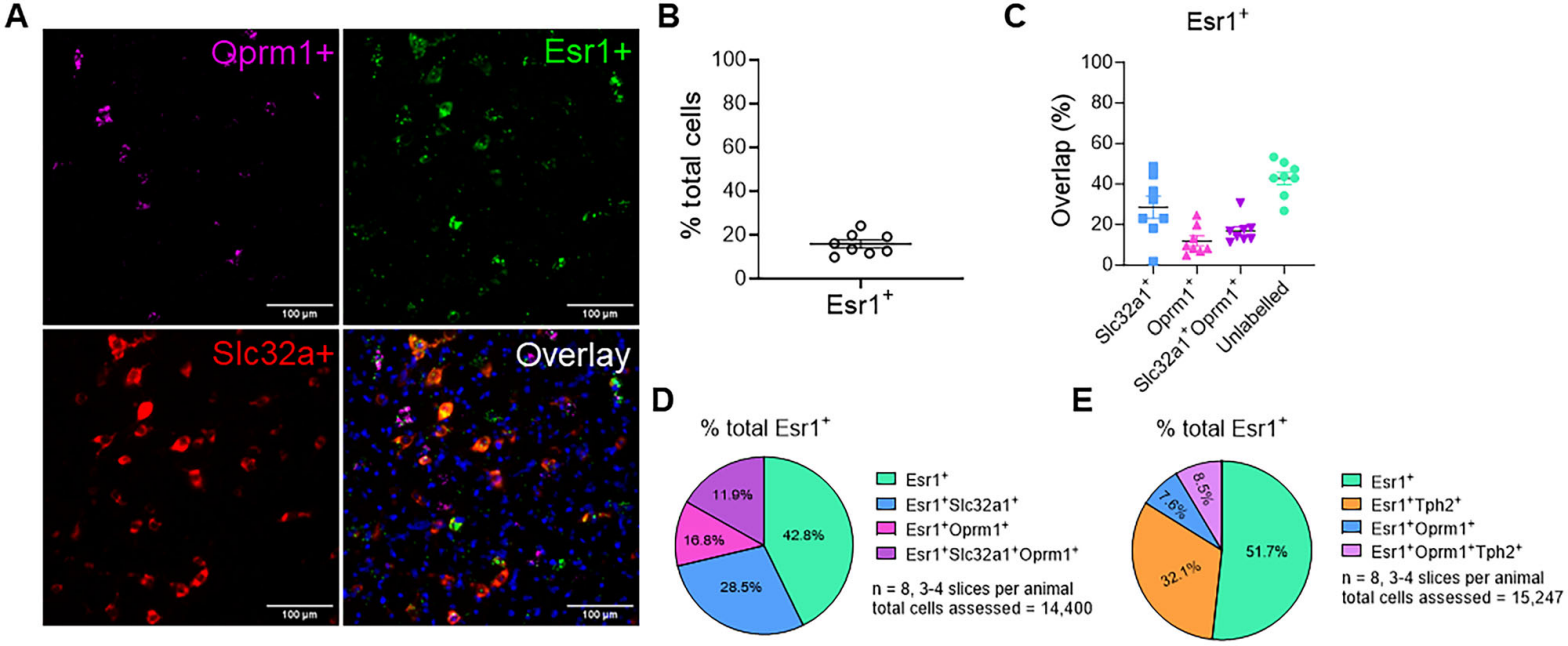
A majority of ER-α is colocalized with VGAT and MOR positive cells. ***A***, Representative image of RNAscope analysis of *Esr1* gene with other genes. ***B***, Percentage of total cells in the RVM expressing *Esr1*. ***C***, RNAscope analysis of *Esr1*-expressing cells in the RVM relative to other markers of interest. Data are mean ± SEM with dots representing individual mice. ***D***, Pie graph showing average of *Esr1-*expressing cells in the RVM relative to cells containing *Slc32a* and/or *Oprm1*. ***E***, Pie graph showing average of *Esr1-*expressing cells in the RVM relative to *Oprm1* and/or *TPH2*.

To determine if ER-α cells are located in the RVM and what potential cell types express this receptor, we used RNAscope to characterize cells containing ER-α (*Esr1*). Specifically, we examined for expression of ER-α in inhibitory neurons by colocalizing with the GABA vesicular transporter (VGAT/*Slc32a1*), in pain facilitation cells, i.e., ON cells (Barbaro et al., 1986), by colocalizing with the μ-opioid receptor (MOR/*Oprm1*), and in serotonergic cells by colocalizing with tryptophan hydroxylase 2 (*Tph2*; Fig. 5). Figure 5*A* shows a representative image of ER-α colocalization with VGAT/*Slc32a1* and MOR/*Oprm1*. Overall, the RNAscope analysis found expression of ER-α in ∼20% of all cells in the RVM (Fig. 5*B*). Of these ER-α positive cells, 28.5% were *Slc32a1*+, 11.9% were *Oprm1*+ (MOR), and 16.8% were both *Slc32a1*+ (VGAT) and *Oprm1*+ (MOR; Fig. 5*C*,*D*). This indicates that ER-α is expressed in inhibitory cells in the RVM. Interestingly this analysis found a large proportion (42.8%) of ER-α positive cells did not express labeling with these two molecular markers suggesting expression in other cell classes. To further identify these cells, we completed another experiment using tryptophan hydroxylase 2 (*Tph2*) as a marker for serotonergic cells which are highly expressed in the RVM. Approximately 32.1% (SEM = 7.53) of *Esr1*-positive cells were colocalized with *Tph2* (Fig. 5*E*); however, there was a large variability between animals (11.85–72.98%). Overall, our analysis shows that ER-α is expressed heterogeneously throughout the RVM in both inhibitory and serotonergic cells.

## Discussion

The current study showed that pharmacological inhibition of aromatase and ER-α, but not ER-β, after induction of activity-induced muscle pain results in the spread of hyperalgesia to the contralateral limb suggesting that aromatization of testosterone to estradiol and activation of ER-α in the RVM protect against widespread pain in male mice. Further, mice with genetic knockdown of ER-α in the RVM prior to induction of the pain model developed both ipsilateral and contralateral hyperalgesia. However, when testosterone was systemically reduced prior to pain, there were no changes in GABA_A_R subunits, vesicular GABA transporter (VGAT/*Slc32a1*), μ-opioid receptor (MOR/*Oprm1*), glutamate ionotropic receptor NMDA type subunit 1 (NR1/*Grin1*), and κ-opioid receptor (KOR/*Oprk1*) mRNA expression in the RVM. Lastly, our molecular characterization of ER-α showed it is contained in a heterogeneous population of cells including inhibitory (VGAT), serotonergic (TPH2), and pain facilitatory (ON-cells; MOR), suggesting a potential role of these neuronal cell populations in activity-induced muscle pain.

In male mice, both locally synthesized and exogenous estradiol can protect and reduce nociceptive behavior. Microinjection of estradiol into the RVM prevents formalin-induced spontaneous pain-related behaviors in male mice (Khakpay et al., 2017), while decreasing local synthesis of estradiol with aromatase inhibitors in the thalamus increases neuropathic pain-induced escape avoidance behaviors (Kramer et al., 2018). Similarly, in naive mice, peripherally administered aromatase inhibitors produce acute intraplanar mechanical hypersensitivity (Fusi et al., 2014). The opposite effects are observed in the spinal cord for the role of local estradiol in modulating pain behaviors. Inhibiting estradiol synthesis with aromatase inhibitors or blocking ERs in the spinal cord reduces formalin-induced spontaneous pain-related behaviors in male rats (Zhang et al., 2012). As in male rodents, spinally applied aromatase inhibitors in females increases mechanical sensitivity after spinothalamic tract lesions, while supraspinal administration decreases sensitivity to visceral pain (Ghorbanpoor et al., 2014; Gao et al., 2017). In female mice, the current study shows inhibition of aromatase has no effect on existing hyperalgesia in the activity-induced pain model. Together, these data suggest local production of estradiol and activation of its receptors can either inhibit or produce hyperalgesia depending on the site (e.g., spinal vs supraspinal), animal pain model (e.g., visceral vs muscle), and sex (male vs female).

In uninjured animals, aromatase inhibitors also have mixed results on pain behavior. In naive male and female rodents, aromatase inhibitors induce mechanical hypersensitivity (Fusi et al., 2014; Robarge et al., 2016). In contrast, aromatase inhibition in spinal cord from male Japanese quails reduces pain behaviors, a response prevented by activation of ERs (Evrard and Balthazart, 2004; Evrard, 2006). Interestingly, women taking aromatase inhibitors for breast cancer treatment often show increased musculoskeletal pain (Henry et al., 2008), suggesting that inhibiting local synthesis of estradiol systemically may produce increased nociception. The current analysis only examined the role of this pathway after activity-induced pain; therefore, it is unclear if blockade of aromatase or ERs in the RVM produces hyperalgesia in naive mice. Our prior study, however, showed removal of testosterone though gonadectomy did not change MWTs prior to injury in male mice (Lesnak et al., 2020). This is consistent with the current study which showed no changes in MWTs after genetic removal of ER-α in the RVM of male mice. This suggests that testosterone aromatization to estradiol in the RVM does not modulate pain thresholds in uninjured animals.

Our pharmacological inhibition studies show an effect within 30 min of administration which reversed before 2 h suggesting that in the RVM, aromatase provides a local source of estradiol that rapidly regulates nociceptive signaling via ER-α. Similarly, aromatase inhibitors produced rapid behavioral changes when administered intrathecally in male Japanese quails and intraplanar in male rats (Evrard, 2006; Fusi et al., 2014). This rapid synthesis of neurosteroids and ER-α activation allows for temporal and regional variability but has not been well characterized in vivo. In rat cultured dorsal root ganglia (DRG) cells, 5 min estradiol exposure inhibits ATP-induced intracellular calcium concentration and currents, mediated by ER-α (Chaban et al., 2003; Chaban and Micevych, 2005). Further, estradiol can activate second-messenger cascades such as phosphatidylinositol 3-kinase/Akt in cultured neurons, protein kinase A and C pathways in hypothalamic neurons, and extracellular signal-regulated protein kinase 1 and 2 in explants of the cerebral cortex (Kelly et al., 1999; Singh et al., 1999; Honda et al., 2000). However, these findings are based on exogenous estradiol application and do not reflect the effects of rapidly synthesized estradiol in vivo (Lee et al., 2002; Chaban et al., 2003). ER-α and ER-β were traditionally thought to mediate the slow, genomic changes, while GPER mediates the rapid, nongenomic signaling (Kumar et al., 1987; Evans, 1988; Carson-Jurica et al., 1990). However, recent evidence suggests that ER-α and ER-β can also perform nongenomic functions (Lokuge et al., 2010; Majumdar et al., 2019). Thus, future studies should examine whether the rapid effects observed in the current study are occurring through genomic or nongenomic mechanisms.

Estradiol and aromatase can regulate inhibitory neurotransmission by changing expression of GABA receptor subunits, VGAT and GABA synthesis enzymes. For example, estradiol increases GABA_A_R α2 and γ1 subunit mRNA expression in the medial preoptic nucleus and the bed nucleus of the stria in female rats (Herbison et al., 1995; Herbison and Fénelon, 1995). Further, VGAT expression is decreased by aromatase inhibition in male rats (Kramer et al., 2018) and fluctuates across the estrous cycle in female rats, likely through binding to estrogen response element sites located on the promotor region of VGAT gene (Ottem et al., 2004; Hudgens et al., 2009; Noriega et al., 2010; Umorin et al., 2016). These data suggest that conversion of testosterone to estradiol could increase inhibition by increasing expression of VGAT or GABA_A_R subunits which could subsequently decrease pain. However, the current study showed no changes in mRNA expression of VGAT or GABA_A_R subunits after removal of testosterone in male mice. It is possible that activation of ER-α produces post-translational modifications of GABA or other inhibitory or excitatory receptors. For example, in animal models of widespread pain, there is increased phosphorylation of the NR1 subunit of the NMDA receptor, and removal of the NR1 subunit in the RVM reduces widespread hyperalgesia; however, there were no changes in phosphorylated NR1 in male or female mice in this model (Da Silva et al., 2010a,b; Gregory et al., 2013). Future studies could explore whether males with reduced testosterone have changes in NR1 subunit in the RVM. Understanding the precise mechanism through which locally synthesized estradiol in the RVM protects male mice against pain remains a subject of further investigation.

The current study shows for the first time expression of ER-α mRNA in the RVM in mice. In contrast, in vivo autoradiography and immunohistochemical analysis indicated low to absent expression of ER-α in the RVM of adult ovariectomized female mice (Mitra et al., 2003; Merchenthaler et al., 2004); however, expression has not been previously explored in males. We further showed that ER-α appears to be distributed heterogeneously among different cell populations in the RVM with 32.1% TPH2+ (serotonergic), 11.9% VGAT+ (inhibitory), 28.5% MOR+ (facilitate nociception), and 16.8% expressing markers for both VGAT and MOR.

It is well accepted that ON cells, which facilitate pain, express MOR with 2/3 of MOR+ coexpressing VGAT+ (GABA; Francois et al., 2017). Neurons in the RVM containing serotonin are often referred to as neutral cells, however, a subset of spinally projecting MOR-positive cells also express serotonin (Potrebic et al., 1994; Marinelli et al., 2002). This suggests that ER-α may contribute to nociceptive processing through multiple neuronal cell types in the RVM. In contrast to the current data, ER-α protein expression shows more specificity in the superficial laminae of the spinal cord with high expression in excitatory cells (VGLUT2) that coexpress substance P or calretinin and very few inhibitory cells (GAD67; Tran et al., 2020). Similarly, in different regions of the limbic forebrain, ER-α receptors are highly expressed on GABAergic neurons (Herbison et al., 1995; Cheong et al., 2015). Therefore, in the RVM, ER-α expression occurs in a heterogeneous manner and may have diverse roles on modulation of pain.

In summary, the current study shows novel evidence of aromatization of testosterone to estradiol within the RVM in protecting against the development of widespread muscle pain in male but not female mice. These effects are mediated through activation of ER-α which is expressed in multiple cell types in the RVM. While these findings in the RVM are unique, there are some limitations to this study including the use of a single behavioral test, and the use of two separate RNAscope experiments to investigate ER-α localization. Future studies should examine the sex-dependent effects of ER antagonism and aromatase inhibition in the RVM in other pain conditions or in naive animals to determine if this is unique to activity-induced muscle pain or more generalizable. Further, it is necessary to examine the potential intracellular mechanisms involved with this local estradiol activity such as NMDA receptor phosphorylation.

## Synthesis

Reviewing Editor: Jennifer Dulin, Texas A&M University Decisions are customarily a result of the Reviewing Editor and the peer reviewers coming together and discussing their recommendations until a consensus is reached. When revisions are invited, a fact-based synthesis statement explaining their decision and outlining what is needed to prepare a revision will be listed below. The following reviewer(s) agreed to reveal their identity: NONE. Note: If this manuscript was transferred from JNeurosci and a decision was made to accept the manuscript without peer review, a brief statement to this effect will instead be what is listed below.

In the opinion of the reviewing editor, the authors have satisfactorily addressed all of the previous reviewers' concerns.
