# Supplementary figures and images for: Local Synthesis of Estradiol in the Rostral Ventromedial Medulla Protects against Widespread Muscle Pain in Male Mice
Source: eNeuro. 2024 Aug 27;11(8):ENEURO.0332-24.2024. doi: 10.1523/ENEURO.0332-24.2024 (PMC11360981; doi:10.1523/ENEURO.0332-24.2024)

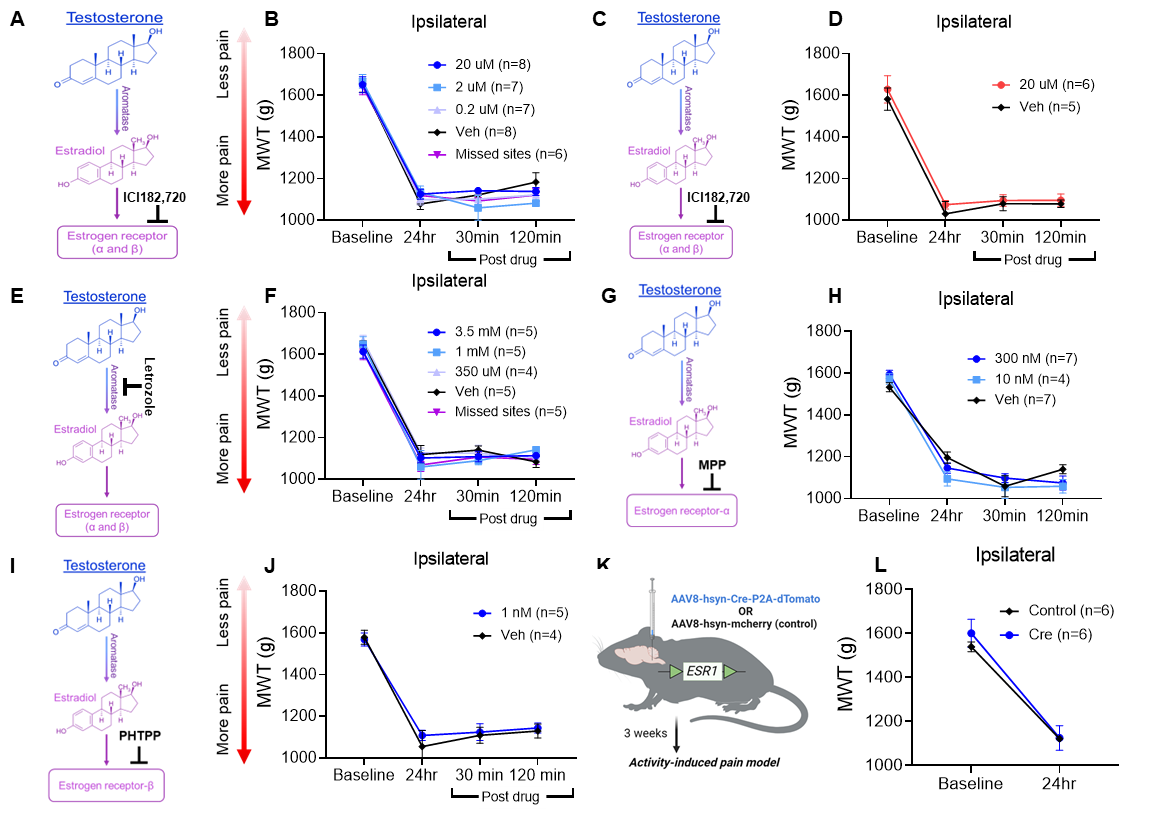

Supplement: Figure 2-1 — Inhibition of ERs, estradiol synthesis, ER-α, or ER-β in the RVM or genetic deletion of ER-α has no impact on ipsilateral hyperalgesia. Download Figure 2-1, TIF file. [file eneuro-11-ENEURO.0332-24.2024-s001.tif]
